# Mosquito vector‐associated microbiota: Metabarcoding bacteria and eukaryotic symbionts across habitat types in Thailand endemic for dengue and other arthropod‐borne diseases

**DOI:** 10.1002/ece3.3676

**Published:** 2017-12-27

**Authors:** Panpim Thongsripong, James Angus Chandler, Amy B. Green, Pattamaporn Kittayapong, Bruce A. Wilcox, Durrell D. Kapan, Shannon N. Bennett

**Affiliations:** ^1^ Department of Microbiology Institute of Biodiversity Sciences and Sustainability California Academy of Sciences San Francisco CA USA; ^2^ Department of Tropical Medicine Medical Microbiology, and Pharmacology University of Hawai'i at Manoa Honolulu HI USA; ^3^ Department of Microbiology University of Hawai'i at Manoa Honolulu HI USA; ^4^ Center of Excellence for Vectors and Vector‐Borne Diseases and Department of Biology Faculty of Science Mahidol University at Salaya Nakhon Pathom Thailand; ^5^ Global Health Asia and Integrative Research and Education Program Faculty of Public Health Mahidol University Bangkok Thailand; ^6^ Department of Entomology and Center for Comparative Genomics Institute of Biodiversity Sciences and Sustainability California Academy of Sciences San Francisco CA USA; ^7^ Center for Conservation and Research Training Pacific Biosciences Research Center University of Hawai'i at Manoa Honolulu HI USA; ^8^ Present address: Department of Tropical Medicine Tulane University New Orleans LA USA; ^9^ Present address: Department of Molecular and Cell Biology University of California Berkeley CA USA

**Keywords:** biodiversity, metabarcoding, microbiota, mosquito, vector, vector‐borne diseases

## Abstract

Vector‐borne diseases are a major health burden, yet factors affecting their spread are only partially understood. For example, microbial symbionts can impact mosquito reproduction, survival, and vectorial capacity, and hence affect disease transmission. Nonetheless, current knowledge of mosquito‐associated microbial communities is limited. To characterize the bacterial and eukaryotic microbial communities of multiple vector species collected from different habitat types in disease endemic areas, we employed next‐generation 454 pyrosequencing of *16S* and *18S rRNA* amplicon libraries, also known as metabarcoding. We investigated pooled whole adult mosquitoes of three medically important vectors, *Aedes aegypti*,* Ae. albopictus*, and *Culex quinquefasciatus,* collected from different habitats across central Thailand where we previously characterized mosquito diversity. Our results indicate that diversity within the mosquito microbiota is low, with the majority of microbes assigned to one or a few taxa. Two of the most common eukaryotic and bacterial genera recovered (*Ascogregarina* and *Wolbachia,* respectively) are known mosquito endosymbionts with potentially parasitic and long evolutionary relationships with their hosts. Patterns of microbial composition and diversity appeared to differ by both vector species and habitat for a given species, although high variability between samples suggests a strong stochastic element to microbiota assembly. In general, our findings suggest that multiple factors, such as habitat condition and mosquito species identity, may influence overall microbial community composition, and thus provide a basis for further investigations into the interactions between vectors, their microbial communities, and human‐impacted landscapes that may ultimately affect vector‐borne disease risk.

## 1 INTRODUCTION

Vector‐borne diseases are a major health burden, accounting for 17% of all infectious diseases ([Link]) and almost 30% of emerging infectious diseases in recent times (Jones et al., 2008). The biological properties of the vectors themselves are important in the distribution of vector‐borne infectious diseases, where transmission is a complex ecological process involving not only hosts and parasites but also a range of interacting organisms in the environment. Recent evidence is mounting that the transmission of vector‐borne pathogens can be inhibited by other vector‐associated microbes (Sinkins, 2013). In the past decade, studies have shown that certain microbes associated with insect vectors could be used to mediate the transmission of diseases including those transmitted by mosquitoes (Capone et al., 2013; Cirimotich et al., 2011; Dong, Manfredini, & Dimopoulos, 2009; Mourya, Pidiyar, Patole, Gokhale, & Shouche, 2002; Ramirez et al., 2014; Tchioffo et al., 2013; Xi, Ramirez, & Dimopoulos, 2008), tsetse flies (Pais, Lohs, Wu, Wang, & Aksoy, 2008; Wang, Wu, Yang, & Aksoy, 2009; Weiss, Wang, Maltz, Wu, & Aksoy, 2013), sand flies (Sant'Anna et al., 2014), and ticks (Gall et al., 2016; Narasimhan et al., 2014). The most striking example is the inhibition by bacterium *Wolbachia pipientis* of virus and parasite infection in mosquito vectors (Bian, Xu, Lu, Xie, & Xi, 2010; Blagrove, Arias‐Goeta, Di Genua, Failloux, & Sinkins, 2013; Frentiu et al., 2014; van den Hurk et al., 2012; Hussain et al., 2012; Moreira et al., 2009). Unfortunately, while studies on the application of microbes such as *Wolbachia* to control human disease have advanced, we still lack basic insight into the natural microbial communities associated with vectors, from viruses to bacteria to single‐celled eukaryotes. Given that infectious vector‐borne diseases are increasing in humans (Jones et al., 2008), our goal is to characterize the composition and structure of microbiota in mosquito vectors across landscapes altered by human activities, where differences may influence the biology of vectors, their vectorial capacity, and ultimately the outcome of microbe‐mediated disease control (Hughes et al., 2014).

Living within and upon mosquitoes are numerous, and diverse microscopic life‐forms, including viruses, bacteria, fungi, protozoa and nematodes, collectively referred to as their microbiota. Recent improvements in the exploration of microscopic organisms through the use of metagenomic PCR (Hugenholtz, Goebel, & Pace, 1998; Liles, Manske, Bintrim, Handelsman, & Goodman, 2003) and next‐generation sequencing (Logares et al., 2012) have allowed more accurate and sensitive documentation of these organisms. Several studies have characterized and compared mosquito bacterial microbiota in natural habitats (Akorli et al., 2016; Buck et al., 2016; Gimonneau et al., 2014; Minard et al., 2012, 2014, 2015; Tchioffo et al., 2015; Yadav et al., 2015; Zouache et al., 2010) but few have characterized eukaryotes and in such cases have targeted fungi (Muturi, Bara, Rooney, & Hansen, 2016; Muturi, Ramirez, Rooney, & Dunlap, 2016; Steyn, Roets, & Botha, 2016). We document mosquito microbiota composition and diversity for vector species *Aedes aegypti*,* Aedes albopictus*, and *Culex quinquefasciatus* across ecologically distinct, yet geographically proximate, habitats in Thailand where many mosquito‐borne diseases circulate. Significant diseases in the area vectored by *Aedes* include dengue (Limkittikul, Brett, & L'Azou, 2014; Nisalak et al., 2016), chikungunya (Chusri et al., 2014; Wanlapakorn et al., 2014), and Zika (Musso & Gubler, 2016). In addition, *Cx. quinquefasciatus*, amongst other *Culex* spp., locally transmits Japanese encephalitis, an important cause of encephalitis in Thailand (Olsen et al., 2015). In characterizing mosquito species and their diversity across habitats, we have observed that mosquito overall diversity decreased with increasing relative abundance of several medically important invasive mosquito vector species and degree of human habitat modification (Thongsripong et al., 2013). In this study, we describe the microbial communities of these disease vectors in the subset of habitats where they were found in our previous study, that is, in rural, suburban, and urban habitats. We employ *16S* and *18S rRNA* amplicon sequencing, a technique referred to as metabarcoding (Taberlet, Coissac, Hajibabaei, & Rieseberg, 2012), to simultaneously characterize the bacterial and eukaryotic microbiota of vector species‐specific pools of three to 25 individual mosquitoes. This study is an important first step in describing the microbial communities associated with different vector species in different habitats. Our results add to the current knowledge of vector‐associated microbiota, especially the eukaryotes, to help reveal microbial species with the potential to control mosquito‐borne diseases, and to emphasize the need to understand how vector symbiotic microbial communities vary across habitats and may ultimately affect vector‐borne disease transmission.

## 2 MATERIALS AND METHODS

### 2.1 Mosquito collection and identification

Adult mosquito collection and habitat characterization was described in a previous study (Thongsripong et al., 2013). In short, we collected adult mosquitoes using a combination of trap types (BG sentinel, Mosquito Magnet, CDC light trap, and CDC backpack aspirator) during the rainy season of 2008 from different habitat types ranging from forest to urban in Nakhon Nayok, a small province situated in central Thailand approximately 60 km northeast of Bangkok. Over 83,000 adults mosquitoes were collected as described in the previous study (Thongsripong et al., 2013). Trap collections from each site were transported on dry ice to a local field station. Upon arrival, mosquitoes were sorted immediately and then stored at −20°C for less than one month before they were transported on dry ice to Mahidol University in Bangkok. The female mosquitoes were identified to species using available morphological keys (Rattanarithikul, Harbach, Harrison, Panthusiri, & Coleman, 2007; Rattanarithikul, Harrison, Panthusiri, & Coleman, 2005; Rattanarithikul, Harrison, Panthusiri, Peyton, & Coleman, 2006; Rattanarithikul et al., 2010; Rattanarithikul, Harbach, et al., 2005; Rattanarithikul, Harrison, Harbach, et al., 2006; Thongsripong et al., 2013). Adult females used in this study were not visibly blood‐engorged. We focused on adult females because they can transmit pathogens to vertebrates through blood‐feeding, and therefore, species identification keys have been better developed for females. For each taxon identified, three to twenty specimens were vouchered as references and housed at the Center of Excellence for Vectors and Vector‐Borne Diseases, Faculty of Science, Mahidol University at Salaya, Nakhon Pathom, Thailand.

Female mosquitoes of the same species were grouped according to their site and date of collection. Of the over 100 mosquito taxa collected, whose mosquito community composition varied significantly across habitat (Thongsripong et al., 2013), we subsampled ten mosquito pools of the species *Ae. aegypti, Ae. albopictus*, and *Cx. quinquefasciatus,* restricted to rural, suburban, or urban habitats, in pool sizes of three to 25 depending on their abundance (Table 1), to characterize their microbiota (Table 2).

**Table 1:** List of study sites for mosquito collection and their habitat characterization

Site	Latitude longitude	Distance (km) to site 09	Trapping dates^a^	No of houses in the site	Percentage or site area covered by trees/concrete^b^	Traffic (human/cars)^c^	Trash/clutter^b^	Surrounding area (within 100 m)
Rural—vegetation includes vegetated patches, rice field, orchard, trees, and bushes around houses								
07	N 14°15.827 E 101°11.157	7.28	19 June–20 June	9 Houses	40/10	¼	Medium	Rice field, vegetation patches, human settlements
08	N 14°17.799 E 101°06.884	15.01	20 June–21 June	12 Houses	40/5	6/10	Medium	Rice field, orchard, human settlements
20	N 14°15.510 E 101°07.788	11.17	12 July–13 July	5 Houses	45/5	2/1	Low‐medium	Rice field, orchard, human settlements
Suburban—vegetation includes rice field, tree, and bushes around houses								
18	N 14°12.754 E 101°12.021	2.06	9 July–10 July	18 Houses	50/20	4/15	Medium	Rice field, human settlements
Urban—vegetation includes trees and bushes around houses								
09	N 14°12.362 E 101°13.094	0	22 June–23 June	24 Houses	25/30	48/127	High	Human settlements
10	N 14°11.811 E 101°13.037	1.02	23 June–24 June	32 Houses	35/40	89/561	High	Human settlements
22	N 14°11.899 E 101°12.749	1.06	15 July–16 July	21 Houses	15/65	22/211	Medium‐high	Human settlements

a Trapping was in 2008.

b The estimation of tree cover and amount of trash and clutter were averaged between two same observers in all sites.

c The number of humans and cars that travelled passed/into the study sites were counted at around noon during the weekdays for half an hour.

**Table 2:** Numbers of rRNA gene sequences belong to samples of different host species and habitat types

MID	Habitat (site)	Species	No. of mosquitoes	Total reads	*16S rRNA* reads		*18S rRNA* reads		Ungrouped^c^
					Before^a^	After^b^	Before^a^	After^b^	
1	Urban (site 9 and 22)	*Ae. aegypti*	25	23,922	829	254	22,047	16,273	1,046
2	Rural (site 20)	*Ae. albopictus*	9	32,030	12,502	10,925	18,206	13,911	1,322
3	Suburban (site 18)	*Ae. aegypti*	25	37,561	15,659	13,035	20,403	15,830	1,499
4	Suburban (site 18)	*Cx. quinquefasciatus*	25	3,877	387	313	3,335	2,497	155
5	Rural (site 8)	*Ae. aegypti*	25	40,201	17,764	15,419	20,692	15,025	1,745
6	Rural (site 8)	*Ae. albopictus*	25	36,946	17,455	13,269	17,789	14,657	1,702
7	Rural (site 8)	*Cx. quinquefasciatus*	25	16,963	15,056	12,481	1,185	884	722
8	Rural (site 7)	*Ae. albopictus*	3	32,688	11,729	10,350	19,740	14,605	1,219
9	Urban (site 10)	*Ae. aegypti*	10	39,908	18,257	16,071	20,094	15,289	1,557
10	Urban (site 10)	*Cx. quinquefasciatus*	25	5,509	2,514	2,045	2,765	2,243	230
Ungrouped^c^				3,343	–	–	–	–	–
Total reads				272,948	112,152	94,162	146,256	111,214	11,197

a Number of sequences before going through quality control procedures.

b Number of sequences before going through quality control procedures.

c Number of sequences that did not have the exact matches to MID tags or primer.

### 2.2 DNA extraction from mosquito pools

Mosquitoes were homogenized in 250 μl of 1× phosphate‐buffered saline using Tissue Lyser II (QIAGEN, CA, USA); 1–2 stainless steel beads (5 mm diameter) were added to each sample and trituration performed at 30 Hz for 3 min with one minute on ice halfway through to cool samples. The homogenate was divided, with half (125 μl) frozen at −80°C for future studies, and the other half mixed with 875 μl of TRIzol LS (Invitrogen, CA, USA). RNA was extracted from samples as per manufacturer's instructions. Following RNA extraction and phase separation, total DNA was isolated from the interphase/organic phase using back extraction buffer (BEB; 4 M guanidine thiocyanate, 50 mM sodium citrate, and 1 M Tris free base). In short, 500 μl of BEB was added to the interphase/organic phase, and the solution was vigorously mixed and then incubated at room temperature for 10 min. The solution was then subjected to centrifugation at 12,000 *g* and 4°C for 10 min, and the upper aqueous phase was collected. Next, we added 400 μl of isopropanol and incubated the samples at room temperature for 5 min. The DNA pellet was precipitated from the solution by centrifugation at 12,000 *g* for 10 min at 4°C. Each DNA pellet was washed twice with 75% ethanol, air‐dried, and dissolved in 50–100 μl of 8 mM NaOH. An appropriate amount of 0.1 M HEPES was added to the sample to obtain a neutral solution, and the DNA was stored at −30°C.

### 2.3 Metabarcoding: *16S* and *18S rRNA* amplicon library preparation and pyrosequencing

To characterize the noneukaryotic and eukaryotic microbiota of these mosquitoes, we amplified and sequenced *16S and 18S ribosomal RNA* (*rRNA*) genes, respectively, using titanium chemistry on the 454 Genome Sequencer FLX instrument (Roche, Switzerland). The variable region V3 of the *16S rRNA* gene was amplified with universal primers U341F (5′ CCT ACG GGR SGC AGC AG 3′) and U800R (5′ CCR GGG TAT CTA ATC C 3′) designed to bind all known bacteria and archaea (Wang & Qian, 2009), and a region of the *18S rRNA* gene was amplified with eukaryotic microbial universal primers Euk82F (5′ GAA ACT GCG AAT GGC TC 3′; López‐García, Philippe, Gail, & Moreira, 2003) and Euk516R (5′ ACC AGA CTT GCC CTC C 3′; Diez, Pedros‐Alio, Marsh, & Massana, 2001). Additionally, the forward primers contain primer A sequence (5′ CGT ATC GCC TCC CTC GCG CCA TCA G 3′) followed by a ten‐base molecular identifier (MID) sequence tag (Table S1), while the reverse primer contained primer B sequence (5′ CTA TGC GCC TTG CCA GCC CGC TCA G 3′) followed by a ten‐base MID tag (Table S1). The 50 μl PCR reaction contained 5 μl of 10× FastStart High Fidelity Reaction Buffer, 200 μM of each dNTP, 0.4 μM of each primer pair, 5% DMSO, and 2.5 U of FastStart High Fidelity Enzyme Blend (Roche, Switzerland). The PCR conditions in most experiments were 94°C for 3 min, 35 cycles at 94°C for 30 s, 50°C (for the *16S rRNA* primer pair) or 54°C (for the *18S rRNA* primer pair) for 30 s, and 72°C for 45 s, followed by 72°C for 8 min. In cases where the samples could not be amplified under the former conditions, 10% DMSO, an annealing time of 45 s, and 40 cycles were used instead.

Amplicons were purified using PCR purification kits following the manufacturer's protocol (NucleoSpin ExtractII; Macherey‐Nagel, Germany). We measured the purified DNA concentration and distribution of amplicon length using the Agilent Bioanalyzer 2100 instrument and Agilent DNA 1000 reagent and kit (Agilent Technologies, CA, USA). Amplicons were combined based on DNA concentration in equimolar ratios. Pyrosequencing was carried out on one‐fourth of a PicoTiterPlate (PTP) using primer B (producing reverse reads; R2).

### 2.4 Sequence quality control and generation of operational taxonomic units

To characterize the number of distinct microbial taxa present in each sample, that is, species richness, we grouped sequence reads into the same operational taxonomic unit (OTU) if they were equal to or less than 3% divergent, a standard threshold which buffers against overestimation of microbial diversity (Kunin, Engelbrektson, Ochman, & Hugenholtz, 2010). Despite a relatively low error rate, the large number of reads resulting from next‐generation sequencing can include a substantial number of erroneous sequences and thus an overestimation of total OTUs (Gilles et al., 2011). Consequently, it is critical to distinguish true sequence diversity from noise introduced by experimental procedures. We adopted strict filtering and quality control procedures prior to the analysis steps in order to limit noise due to sequencing errors (described below). We utilized a sequence analysis pipeline: QIIME, http://qiime.org (Caporaso, Kuczynski, Stombaugh, & Bittinger, 2011) for sequence processing and OTU generation. The command lines used in the analyses are given in Figure S1. Default settings were used with the exception of minor changes described below.

Analysis began with assignment of reads to samples based on MIDs followed by initial sequence quality check. Suitable quality includes (i) no mismatches to either MID and reverse primer sequences, (ii) meeting the mean quality score for sequences, (iii) sequence length of at least 200 bp, (iv) no ambiguous called bases, and (v) no homopolymers over 9 bp. We did not discard entire sequence reads containing low‐quality score sections, which would have resulted in too few remaining sequences; instead, those sequences were truncated to exclude the poor‐quality section. After initial quality check, we then employed QIIME's algorithms that approximate PyroNoise (Quince et al., 2009; Reeder & Knight, 2010) to cluster the flowgram (analogous to the trace data in Sanger sequencing) and denoise the data. Next, we removed forward primer sequences from the end of reads, allowing at most one mismatch to the primer sequence, again to minimize the number of discards, where reads with more than one mismatch to the forward primer sequences were removed. Base calling also becomes less accurate toward the end of the reads (Ledergerber & Dessimoz, 2011). After the quality filtering processes, sequences were aligned (Caporaso et al., 2010) and grouped into OTUs based on ≤3% divergence. Specifically, within this step, we employed “pick_otus.py” command in QIIME to cluster reads de novo and maintained the default options, including the use of UCLUST as the OTU picking method (Edgar, 2010). Next, the OTUs identified as chimeric by the Chimera Slayer algorithm (Haas et al., 2011) were removed. There was no chloroplast or mitochondrial sequences present after this step.

### 2.5 Alpha‐diversity analysis

As diversity indices are sensitive to sample size, in this case number of reads analyzed (Magurran, 1988), we used two approaches. First, rarefaction analysis was used to project the rate of accumulation of OTUs with the addition of more sequence reads, resulting in rarefaction curves. Second, equal numbers of reads were randomly subsampled from each original set of reads prior to OTU estimation. Samples with fewer than 25% of the average number of reads were excluded, resulting in approximately 7,100 and 11,000 reads for analysis of *16S* and *18S rRNA*, respectively. This set of OTUs was used to calculate the species diversity indices Shannon entropy (Shannon, 1949) and diversity estimator Chao1 (Chao, 1984) for across‐sample comparisons.

### 2.6 Beta‐diversity analysis

To assess beta‐diversity, indicative of the intersample differences between mosquito microbiota, we calculated the normalized weighted UniFrac distances (Lozupone & Knight, 2005) of all sample pairs using Fast UniFrac (Hamady, Lozupone, & Knight, 2009). The input phylogenetic trees for Fast UniFrac were constructed using FastTree (Price, Dehal, & Arkin, 2009) which infers approximately maximum‐likelihood tree with the GTR + CAT model. The taxa that we used as outgroups were *Thermotoga maritima* and Chordata sequences (*Homo sapiens sapiens* and *Mus musculus*) for *16S* and *18S rRNA* tree, respectively. We then applied the Unweighted Pair Group Method with Arithmetic Mean (UPGMA) algorithm to identify clusters based on these calculated pairwise UniFrac values. Finally, we performed jackknife resampling analysis of sample clusters to assess the confidence in the nodes of the UPGMA tree. The number of permutations was 100, and we specified that approximately 75% of the number of smallest sample should be kept in the analysis during each jackknife replicate.

### 2.7 Determination of mosquito microbiota composition

To determine the taxonomic identities of OTUs, we used QIIME's command line “assign_taxonomy.py” and maintained the default options (Wang, Garrity, Tiedje, & Cole, 2007). We used the Greengenes database for *16S rRNA* sequence analysis (DeSantis et al., 2006) and SILVA taxonomy sequence database for *18S rRNA* analysis (Yilmaz et al., 2013). Note that although *16S* primers were designed to amplify both bacteria and archaea, no archaea were recovered. Archaea have no known associations with mosquitoes (Clemens, 2012), associating primarily with wood and detritus feeders such as termites and beetles (Engel & Moran, 2013). Results based on *16S* will herein address bacterial diversity.

## 3 RESULTS

### 3.1 Sequence processing

A total of 273,590 DNA sequence reads of the *16S* and *18S rRNA* genes were generated from the 10 mosquito‐pool samples, varying in number by host species and habitat type (Table 2). Read numbers were not equally distributed across samples despite standardizing DNA input into library preparation, which may arise due to differential inefficiencies in the emulsion PCR (Kanagal‐Shamanna, 2016) for different microorganisms or inaccurate DNA quantification resulting from unknown impurities (Binladen et al., 2007). The average number of raw *rRNA* sequence reads per sample was 26,961 (min = 3,877; max = 40,201) with an average length of 423 bp. A total of 14,540 reads were discarded due to mismatching MID and primer sequence tags, resulting in averages of 11,215 reads (95% CI = ±4,473; min = 387; max = 18,257) and 14,626 reads (95% CI = ±5,279; min = 1,185; max = 22,047) per sample for *16S* and *18S rRNA* genes, respectively.

After sequence quality control, there were a total of 94,162 *16S rRNA* reads (67% of the original number of reads) with an average length of 415 bp and 111,214 *18S rRNA* reads (76% of the original number of reads) with average read length of 448 bp. *16S* and *18S rRNA* reads were assigned to 424 (283 centroids, 141 singletons) and 192 (170 centroids, 22 singletons) read sets, respectively. These sets of reads were used in further diversity analyses.

### 3.2 Richness of microbiota associated with mosquito vector species

**Figure 1:**
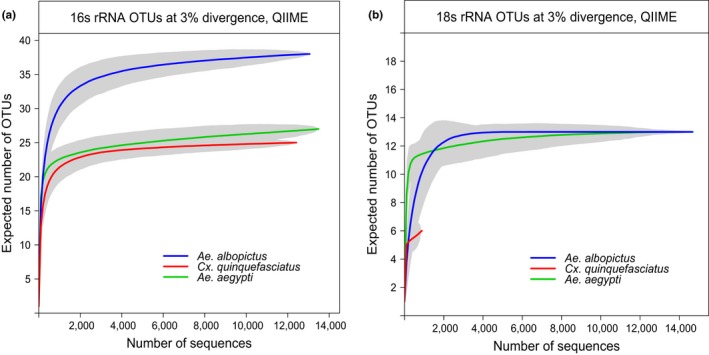
*16S and 18S rRNA* OTU richness of mosquito‐associated microbial communities collected from rural habitats (a and b, respectively). Rarefaction curves by vector species in the rural habitat where all species were collected for pool sizes of 25. Gray shading around lines represents the 95% confidence interval of the estimate

The total number of bacterial OTUs found in all samples was 90, and the median estimates of bacterial OTUs per sample were 18. For eukaryotic microbiota, the total estimated OTUs were 53, and the median estimate of OTUs was 12.5. Rarefaction curves for *16S rRNA* and *18S rRNA* from each vector species are shown in Figure 1 for one habitat where all three vectors had adequate numbers of sequences for comparison (rural, Table 1). *Aedes albopictus* shows the highest OTU richness according to *16S rRNA* data (Figure 1a). OTU richness of *18S rRNA* gene sequences was similar in all vector species that had adequate numbers of sequences (Figure 1b). The numbers of observed bacterial and eukaryotic OTUs in all pooled mosquito samples are shown in Table S2.

### 3.3 Richness of microbiota associated with mosquito vectors by habitat type

**Figure 2:**
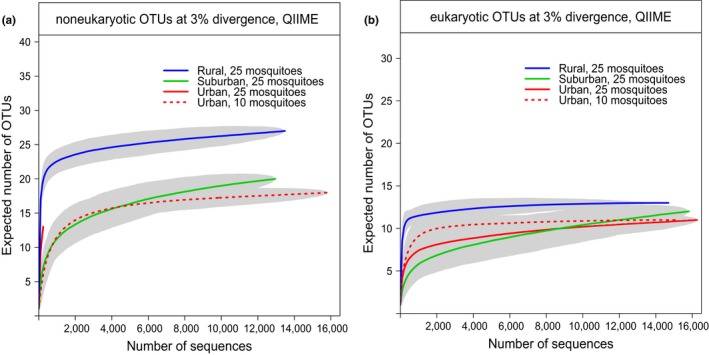
*16S and 18S rRNA* OTU richness of *Aedes aegypti*‐associated microbial communities across habitat type. The expected numbers of bacterial (a) and eukaryotic (b) OTUs associated with *Ae. aegypti* collected from different habitats were plotted as a function of sampling effort (in this case, number of sequences). Gray shading represents the 95% confidence interval of the estimate

In this study, the bacterial microbiota of *Ae. aegypti* had a higher richness in rural than in suburban habitats based on rarefaction curves of OTUs from *16S rRNA* reads for the same pool size of 25 (Figure 2a). OTU richness appears to be lowest in the urban habitat according to pool‐of‐10 estimates, but direct comparison for the same pool size cannot be made as the pool‐of‐25 urban estimate is uncertain due to a lack of reads (Table 2). The eukaryotic microbiota of *Ae. aegypti* also appears to be slightly more OTU‐rich for a given pool size (25) in rural habitats than in suburban and urban (Figure 2b). Across‐habitat comparison of OTU richness for *Cx. quinquefasciatus* samples was not possible due to insufficient sequence recovery, nor for *Ae. albopictus* samples which were only collected in the rural habitat.

Pool size did affect OTU richness estimates of bacteria by rarefaction analysis, where the larger the pool size, the higher the richness estimated. Rarefaction curves based on *16S rRNA* for *Ae. albopictus* samples all from the rural habitat but differing in pool sizes (3, 9, and 25 mosquitoes) projected higher OTU richness with increasing pool size (Figure S2a). This trend was not consistently observed for estimates of eukaryote richness (Figure S2b).

### 3.4 Diversity of microbiota associated with mosquito vectors

**Figure 3:**
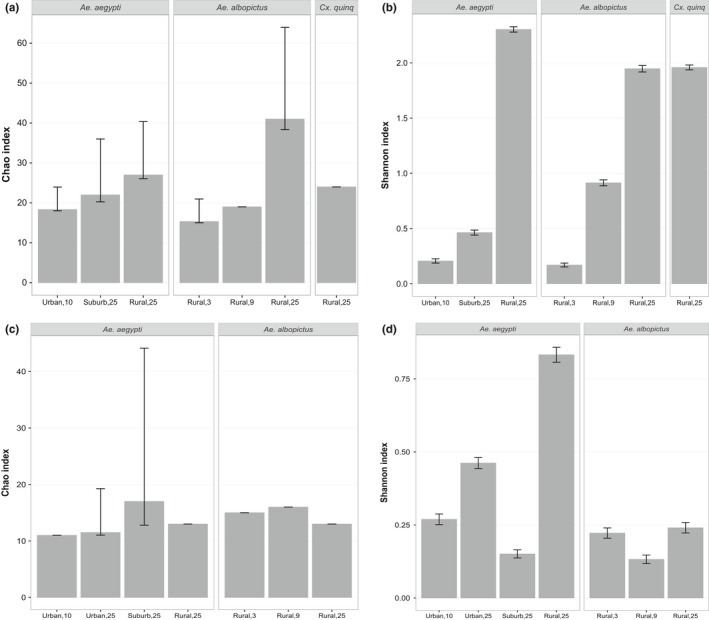
Diversity indices (Chao1 and Shannon) for mosquito‐associated microbiota. To assess the alpha‐diversity of mosquito‐associated bacterial (a and b) and eukaryotic (c and d) communities, diversity indices including the Chao1 (a and c) and Shannon entropy (b and d) were calculated from *16S* and *18S rRNA* gene‐derived OTUs defined at 3% genetic divergence, equalized for sample size. Error bars show 95% confidence intervals

Diversity, a function of not only species richness (number of species) but also species relative abundance, here measured with the diversity indices Shannon entropy (Shannon, 1949), and Chao1 (Chao, 1984), varied greatly by sample (Figure 3). Chao indices of diversity ranged from 11 to 41 with an average of 19 OTUs across samples. The Shannon indices of diversity ranged from 0.13 to 2.30 with an average of 0.73.

In this study, diversity indices suggest that bacteria increase in diversity from the samples collected in the urban to rural sites for a given vector species, in this case *Ae. aegypti* (Figure 3a,b). Bacterial diversity associated with *Ae. albopictus* from rural sites also increased with pool size (Figure 3a,b). Diversity indices based *18S rRNA* were inconsistent, except for the Shannon index, where eukaryote microbial diversity was again highest in the rural site for a given vector species, in this case *Ae. aegypti*, and pool size (25; Figure 3c,d).

### 3.5 Changes in microbiota community across vector species and habitat type

**Figure 4:**
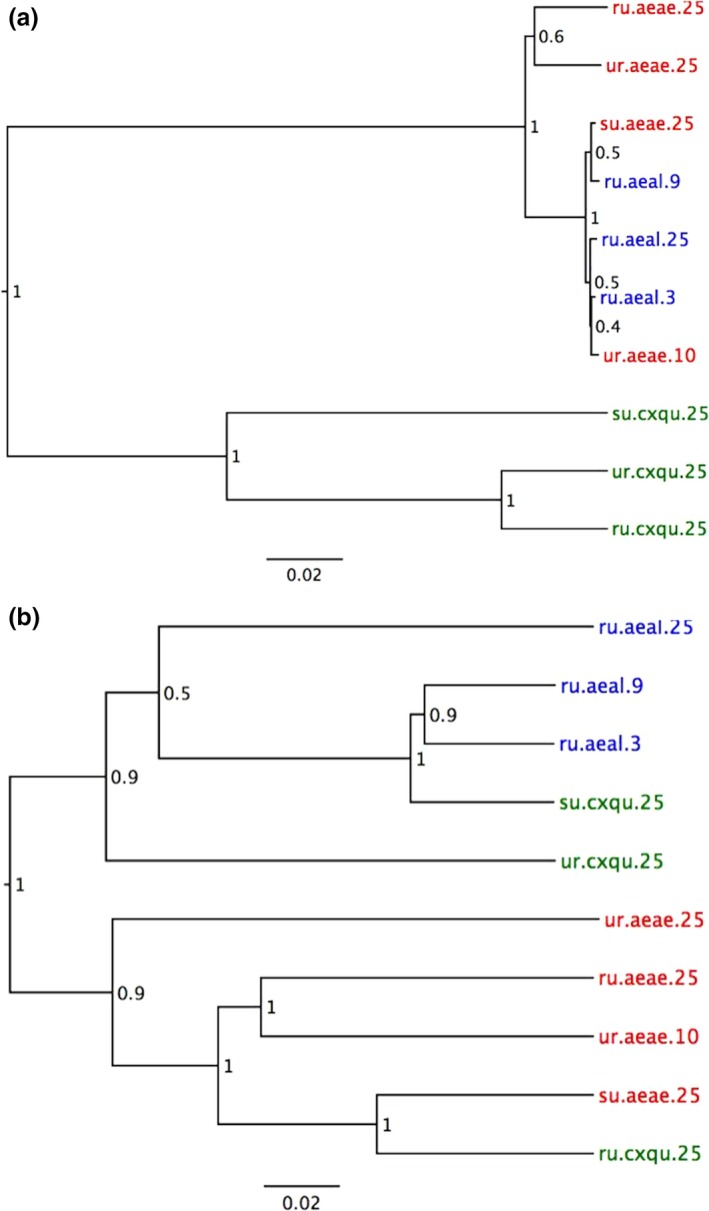
Clustering of samples based on microbiota. Cluster diagrams based on Unweighted Pair Group Method with Arithmetic Mean (UPGMA) of pairwise weighted UniFrac distances show sample relationships based on microbial communities. Jackknife support is shown at the nodes. The clustering‐based bacterial microbiota is shown in (a) and eukaryotic microbiota in (b). Tips are coded for vector species, habitat, and pool size: red for *Aedes aegypti* (aeae), blue for *Aedes albopictus* (aeal), and green for *Culex quinquefasciatus* (cxqu); ru, rural; su, suburban; ur, urban; pool size appended

**Figure 5:**
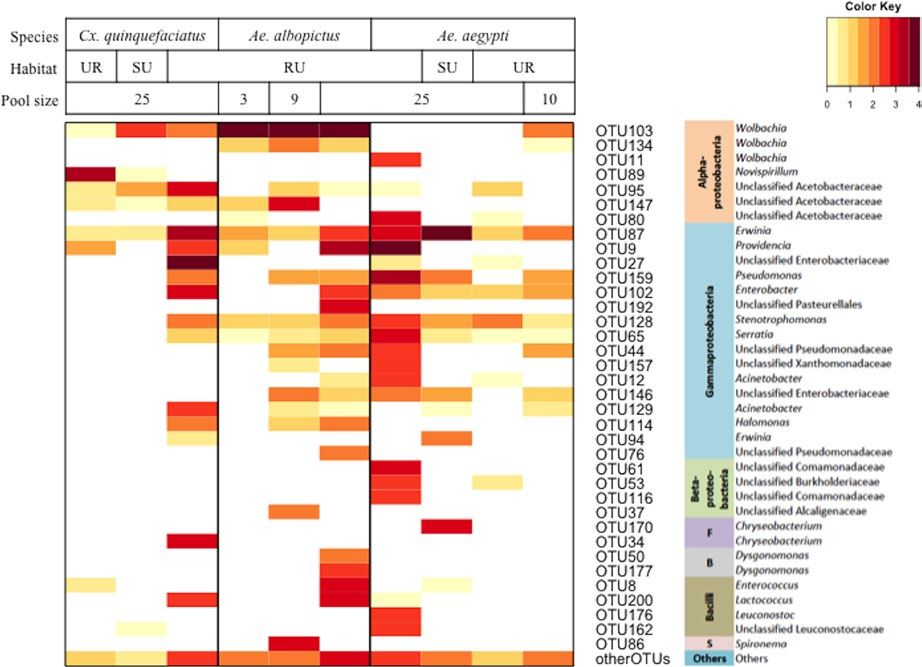
Heatmap showing the composition of mosquito‐associated bacterial microbiota. The OTUs with total number of sequence >150 are presented in rows of the heatmap along with their most likely taxonomy. The rest of OTUs were grouped into “Other OTUs” group at the bottommost row. The color key on the top right corner indicates the powers of the log‐transformed base 10 of the sequence numbers in the cells. S, Spirochaeta; F, Flavobacteria; B, Bacteroidia; RU, rural; SU, suburb; UR, urban. Note that OTU11 is the *Wolbachia* of *Dirofilaria*

Comparison of mosquito microbiota or beta‐diversity across samples using cluster analysis in this study indicated significant differences to a certain extent by both species and the habitats characterized in this study (Figure 4). The grouping of samples by host species was also observed somewhat in Nonmetric Multidimensional Scaling (NMDS) plots at least for bacterial community composition (Figure S3). Bacterial community composition was similar within a given vector species for *Ae. aegypti* and *Ae. albopictus*, but not so in the case of *Cx. quinquefasciatus*, whose microbiota shared similarities with both *Aedes* species rather than being *Culex*‐specific (Figure 4a). Interestingly, sequences identified as *Wolbachia* spp. constituted the majority of *Ae. Albopictus‐* and *Cx. quinquefasciatus*‐derived sequences (Figure 5); when these were excluded, there was no consistent clustering by either vector species or habitat type (Figure S4).

**Figure 6:**
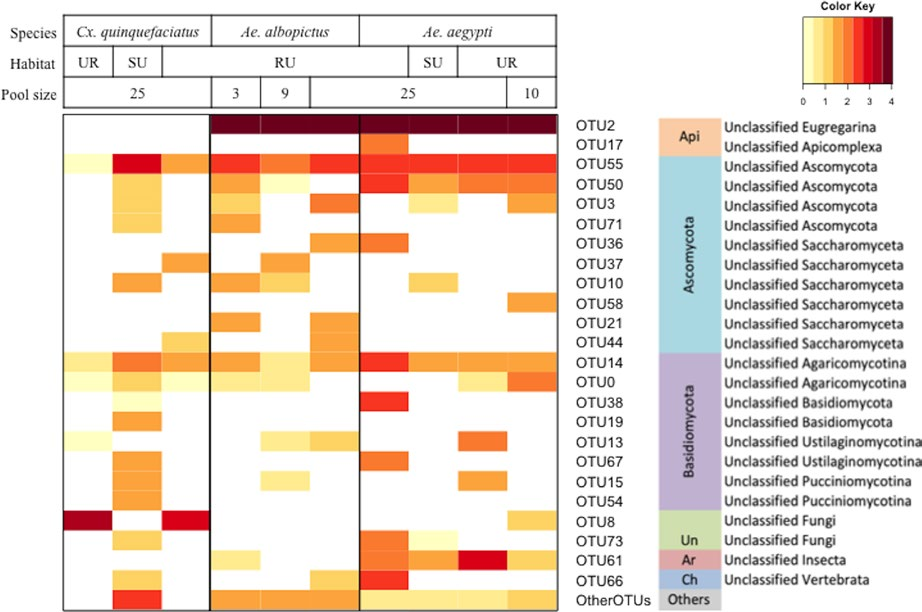
Heatmap showing the composition of mosquito‐associated eukaryotic microbiota. The OTUs with total number of sequence >50 are presented in rows of the heatmap along with their most likely taxonomy. The rest of OTUs were grouped into “Other OTUs” group at the bottom row. The color key on the top right corner indicates the powers of the log‐transformed base 10 of the sequence numbers in the cells. Api, Apicomplexa; Un, Unclassified Fungi; Ar, Arthropoda; Ch, Chordata; RU, rural; SU, suburb; UR, urban

Eukaryotic microbiota clustered by vector genus, with *Cx. quinquefasciatus* exhibiting a more distinct eukaryotic microbiota than the *Aedes* spp., regardless of habitat. When the most common sequences, identified as *Ascogregarina* spp. (Figure 6), were excluded from *Ae. aegypti* and *Ae. albopictus* sample analysis, no clustering pattern based on vector genus, species, or habitat type was observed (Figure S4).

### 3.6 Microbiota composition of the mosquito vectors

The identities of bacterial and eukaryotic OTUs as determined by our database search algorithms implemented in QIIME are summarized to major taxonomic group in Figures 5 and 6 (see Tables S3 and S4 for taxonomic identities and sequence abundance of all OTUs). In total, 16S *rRNA* and 18S *rRNA* sequences found in mosquito pools were identified to at least six phyla of bacteria (from most abundant to least: Proteobacteria, Firmicutes, Bacteroidetes, Spirochaetes, Actinobacteria, and the candidate Phylum TM7) and nine single‐celled eukaryotic phyla (from most abundant to least: Apicomplexa, Ascomycota, Entomophthoromycota, Basidiomycota, Mortierellomycotina, Chytridiomycota, Heterokontophyta, Mucoromycotina, and Mycetozoa). In addition, chordate‐like and arthropod *18S rRNA* sequences were recovered from some samples (Table S4). The chordate‐like sequences are assumed to be bloodmeal‐derived. No Archaea were found in this study.

The dominant bacterial classes across all samples were Gammaproteobacteria, 48.63% of all bacteria, with genera *Erwinia* and *Providencia* the most abundant, and Alphaproteobacteria, 40.97%, with genus *Wolbachia* the most abundant. The most abundant eukaryote averaged across all samples was a group of insect‐specific gregarine symbionts in the phylum Apicomplexa (OTU 2, 4, 17, 22, and 31 amount to 91.09% of all eukaryotic reads, see Table S4). We were not able to identity these OTUs to the generic level within our QIIME analysis pipeline; however, we submitted representative sequences of each OTU (Table S6) to NCBI databases using the BLAST search algorithm and found that most (except OTU 22) belong to *Ascogregarina* spp. (most likely either *Ascogregarina culicis* or *A. taiwanensis*, common potentially parasitic endosymbionts of *Ae. aegypti* and *Ae. albopictus*, respectively). Fungal eukaryotes were also detected, of which the most abundant OTUs were in phylum Ascomycota (3.31% of all eukaryotes) and Entomophthoromycota (2.67% of all eukaryotes).

The microbial composition of samples based on identity of sequences and their relative abundance varied with the vector species investigated in this study, as well as their habitats and pool size for a given vector species (Figures 5 and 6). For instance, the dominant microbial taxon varied between vector species. The dominant taxa of bacteria associated with *Ae. aegypti*,* Ae. albopictus*, and *Cx. quinquefasciatus* were *Erwinia* (47.52%), *Wolbachia* (74.02%), and an unclassified Enterobacteriaceae (28.61%), respectively. The dominant taxon of eukaryotes associated with *Ae. aegypti* and *Ae. albopictus* was the group of *Aedes*‐specific gregarine symbionts in the phylum Apicomplexa (91.55%, 96.77%, respectively), whereas the eukaryotic microbiota of *Cx. quinquefasciatus* was dominated by fungi of Phylum Entomophthoromycotina (51.65%; BLAST results of the representative sequence of this OTU revealed 99% identity to a sequence of *Furia* sp. and 98% identity to sequences of *Pandora* sp.) and Ascomycota (33.55%; BLAST revealed 100% identity to sequences of *Cladosporium* sp.). Within a vector species collected in multiple habitats, microbial communities further differed in terms of most common taxa. The bacteria associated with *Ae. aegypti* collected from urban, suburban, and rural habitats were *Stenotrophomonas* (24.69%), *Erwinia* (91.74%), and *Providencia* (39.00%), respectively. The dominant taxa associated with *Cx. quinquefasciatus* collected from urban, suburban, and rural habitats were *Novispirillum* spp. (96.35%), *Wolbachia spp*. (75.55%), and the unspecified Enterobacteriaceae (33.90%), respectively. Overall, for a given vector species sampled across multiple habitats (*Cx*. *quinquefasciatus* and *Ae. aegypti*), there were more identified taxa of bacteria in rural than in urban and suburban habitats (Figure 5). In terms of habitat variation in eukaryote composition for a given vector, the dominant eukaryote within *Aedes* spp. remained *Ascogregarina* across habitat types; however, the dominant eukaryotes associated with *Cx. quinquefasciatus* varied from fungi of the phylum Entomophthoromycotina in urban (99.73%) and rural sites (74.77%), to the phylum Ascomycota (73.23%) in the suburban site.

Some identifications required further investigation, such as sequences identified as *Wolbachia* spp. within *Ae. aegypti* (Figure 5). *Aedes aegypti* has not been known to harbor *Wolbachia* in nature (Bian et al., 2010) a recent study reported its presence in field‐caught *Ae. aegypti* in the United States (Coon, Brown, & Strand, 2016). In our study, *Wolbachia* was identified in two *Ae. aegypti* pools, one from a rural and one from an urban site. *Wolbachia* sequences from the rural *Ae. aegypti* pool were most closely related to a *Dirofilaria*‐infecting species (Figure S5). *Dirofilaria* are parasitic roundworms infecting certain mammals by way of mosquito vectors including *Ae. aegypti* (Simón et al., 2012). *18S rRNA* sequences resembling *Dirofilaria* were also recovered from the same pool, although the sequence was not of sufficient quality to be represented in the final taxon list (data not shown, but *Dirofilaria* sp. sequence accession number is MF 319764). Several other sequences of *Wolbachia* were found in the *Ae. aegypti* pool sampled from the urban site as well as in pools of the other mosquito species in this study (Figure 5). Although a known endosymbiont of other mosquito species, *Wolbachia* has only been detected in *Ae. aegypti* in nature in one other study (Coon et al., 2016). Reads identified as *Wolbachia* recovered from the *Ae. aegypti* pools were not identical to reads identified as *Wolbachia* in any of the other pools in the study (Figure S4), suggesting that they were not the result of sample contamination from another vector species or pool. To further address whether the *Wolbachia* sequences were contaminants from accidental inclusion of non‐*Ae. aegypti* material in the pool, we screened *18S rRNA* sequences for non‐*Ae. aegypti* mosquito sequences and only *Ae. aegypti* sequences were recovered. We also amplified, cloned, and sequenced mosquito *COI* sequences from the pool and confirmed that all 30 clones examined were *Ae. aegypti* (data not shown). Thus, our findings suggest the possibility that some form of *Wolbachia* may associate naturally with *Ae. aegypti* and that further study is merited.

## 4 DISCUSSION

This study contributes significantly to our knowledge of mosquito microbiota in nature, by employing culture‐independent amplicon‐based metagenomic deep sequencing to characterize *both* bacterial and eukaryotic microbial rRNA sequences associated with field‐collected mosquito vectors of disease, *Ae. aegypti, Ae. albopictus*, and *Cx. quinquefasciatus*. Previous studies mainly focused on noneukaryotic microbes in the midgut using either culture‐dependent or culture‐independent methods (Minard, Mavingui, & Moro, 2013). Those that applied the culture‐independent deep sequencing approach were mainly restricted to gut bacterial communities (Minard et al., 2012; Wang, Gilbreath, Kukutla, Yan, & Xu, 2011) or other isolated adult arthropod parts (Damiani et al., 2010; Sharma et al., 2014), although one study investigated bacteria diversity from whole *Ae. albopictus* (Minard et al., 2014). Studies using shotgun metagenomic sequencing (which is agnostic to the genetic material sequenced, as opposed to the gene‐specific amplicon‐based method used here) have identified both bacterial and eukaryotic microbes associated with mosquitoes, however with insufficient depth to perform diversity analyses (Chandler, Liu, & Bennett, 2015). Thus, our study contributes a uniquely comprehensive view of mosquito‐associated microbial communities, unrestricted to bacteria, whose dynamics across vectors and landscapes could ultimately inform the distribution of infectious diseases.

Overall richness of bacterial OTUs in our study, estimated as 18 OTUs, was much lower than comparable studies using the same OTU cutoff (3% genetic divergence). For example, a median of 42 bacterial OTUs was estimated from the guts of eight mosquito species (Minard et al., 2012); higher still by at least 200 OTUs was the bacterial richness in the gut of a single adult *Anopheles gambiae* (Wang et al., 2011). This enormous variability amongst studies, including ours, likely reflects inherent differences in microbiota diversity, composition, and heterogeneity amongst individuals, due to vector species and site of mosquito collection. In addition, individual mosquitoes can be extremely variable in their gut microbiota as observed by Osei‐Poku, Mbogo, Palmer, and Jiggins (2012) of wild mosquitoes from Kenya. Thus, estimates are extremely sensitive to sample size (both number of individual vectors sampled and numbers of microbes sampled as a function of sequencing depth) and sample processing. Sequence quality filtering to eliminate erroneous base substitutions arising from PCR and/or pyrosequencing had a significant impact on microbial diversity estimates: application of early‐stage denoising filters reduced our estimated numbers of OTUs at 3% divergence by twofold to fivefold (data not shown). We acknowledge that by adopting the most conservative noise reduction protocols, we may lose certain OTUs, such as the *Dirofilaria* reads already mentioned.

Despite differences in OTU richness and diversity estimates across studies, the taxa of microbes commonly associated with *Ae. aegypti, Ae. albopictus*, and *Cx. quinquefasciatus* were consistent with previous investigations (Dada et al., 2014; Minard et al., 2013; Moro, Tran, Raharimalala, Ravelonandro, & Mavingui, 2013; Pidiyar, Jangid, Patole, & Shouche, 2004; Tranchida, Riccillo, Rodriguero, Garcia, & Micieli, 2012; Zouache et al., 2010). The most abundant genus of bacteria in terms of numbers of identified taxa was *Wolbachia*, a common intracellular parasitic bacterium of many insect species including *Ae. albopictus* and *Cx. quinquefasciatus* (Werren, Baldo, & Clark, 2008). Other bacterial taxa, such as *Providencia* and *Erwinia* (all Family Enterobacteriaceae), are commonly identified with *Aedes, Culex*, and other mosquitoes (Demaio, Pumpuni, Kent, & Beier, 1996; Minard et al., 2012, 2014; Rani, Sharma, Rajagopal, Adak, & Bhatnagar, 2009; Zouache, Michelland, Failloux, Grundmann, & Mavingui, 2012). Likewise, the most common eukaryotic microbes identified in this study have also been previously found associated with mosquitoes. For example, an OTU most likely classified to *Cladosporium* sp., which was found in nine of the ten samples, has been found in *Culex pipiens* from California (Chandler et al., 2015).

Interestingly, our results indicate infection with both a eukaryotic and a bacterial pathogen in the urban *Cx. quinquefasciatus* sample. An OTU most likely classified as *Pandora/Furia*, which comprises the majority of the eukaryotic reads in this sample, is a known and exclusive insect pathogen (Gryganskyi et al., 2012). Likewise, *Novispirillum,* which comprises over 96% of the total bacterial reads in this study, has been isolated from unhealthy‐looking *Cx. pipiens* larvae in another study (Tranchida et al., 2012). Notably, experimental inoculation of mosquito larvae with *Novispirillum* only caused mortality when the cuticle was damaged by co‐inoculation with a parasitic nematode or by needle puncture (Tranchida et al., 2012). As entomopathogenic fungi infect the host by penetrating the cuticle (Shah & Pell, 2003), this suggests the interesting scenario that an initial infection by *Pandora/Furia* precipitated a secondary infection by *Novispirillum*. This highlights the importance of characterizing both the bacterial and eukaryotic microbial communities in the same samples.

Another notable microbe/mosquito association recovered in this study included *Wolbachia* in *Ae. aegypti*. We identified *Wolbachia 16S rRNA* sequences in pools of *Ae. aegypti* collected from rural and urban habitats (Figure 5). *Wolbachia* sequences in the rural sample are almost all of the C group and related to strains that infect nematodes of the genus *Dirofilaria* (Figure S5). Given that *Dirofilaria* sequences were also recovered from the same pool, we suggest that these *Wolbachia* recovered from the rural *Ae. aegypti* pool are actually associated with parasitic nematodes found in this vector. Surprisingly, *Wolbachia* sequences identified in the pool of *Ae. aegypti* collected from the urban site grouped with other mosquito‐associated strains including *Culex* spp. and *Ae. albopictus Wolbachia* (Figure S5), but further investigation is needed in order to confirm this observation and to assign the strain of *Wolbachia* to group A or B. Although a known endosymbiont of many insects and other invertebrates, *Wolbachia* has not been detected in *Ae. aegypti* in nature until recently: Coon et al. (2016) discovered *Wolbachia* in a subset of field‐caught *Ae. aegypti* larvae and adults from the southeastern United States, starting with a similar methodology of *16S rRNA* metagenomic sequencing. Their data suggest a past lateral transfer from *Ae. albopictus*, possibly facilitated by the overlapping ranges and habitats of these two mosquito species (Coon et al., 2016). Novel *Wolbachia—*mosquito associations have also been reported in *An. gambiae* collected in West Africa (Baldini et al., 2014).

Given that *Wolbachia* can spread to new populations amongst arthropods within and between species through horizontal transmission (Ahmed, Breinholt, & Kawahara, 2016; Boyle, Oneill, Robertson, & Karr, 1993), its resulting distribution could conceivably be quite heterogeneous across different populations within a species. Despite the preliminary nature of our findings, the implications that *Wolbachia* may naturally occur in some *Ae. aegypti* populations are worth briefly discussing. *Wolbachia* can affect vector competence (Jupatanakul, Sim, & Dimopoulos, 2014) and vectorial capacity by reducing mosquito populations (Atyame et al., 2016). In the former case, dengue virus is blocked in experimentally transfected *Ae. aegypti* with *wMel* and *wMelPop* strains of *Wolbachia* (Hoffmann, Ross, & Rai, 2015): These mosquitoes are being released to suppress dengue transmission (Frentiu et al., 2014). In the latter case, experimentally transfected *Ae. albopictus* have been developed and released into *Ae. albopictus* populations with naturally occurring *Wolbachia* for population suppression (Atyame et al., 2016; Mains, Brelsfoard, Rose, & Dobson, 2016) by way of a mechanism known as cytoplasmic incompatibility (CI), where matings between males and females with different strains of *Wolbachia* experience mortality of the developing embryos (Zabalou et al., 2004). Thus, naturally occurring *Wolbachia* incompatible with introduced transfected strains could interfere with virus suppression and could also lead to local *Ae. aegypti* population suppression. Further study is warranted, not only to confirm results from this study but also, given other records of naturally occurring *Wolbachia* in *Ae. aegypti* (Coon et al., 2016) findings, to delineate the specificity, frequency, geographic breadth, and the disease suppression and vector control implications of this potentially novel vector–microbe association

We recognize that some limitations exist in our study, which affect the generality of our findings and their interpretations. First, because we wanted to maximize the breadth of locations and species in this study for a broad survey, we did not include replications sufficient to statistically test associations by site and species. Similarly, we employed multiple trap types to attract a range of mosquito species, but these also target mosquitoes at different physiological states (e.g., recently bloodfed, resting, or host‐seeking). Although some studies suggest that physiological state may affect the composition and diversity of midgut microbiota (Wang et al., 2011; Yadav et al., 2016), others have demonstrated that *Ae. aegypti* harbors a stable bacterial community during its adult life (David, dos Santos, Vicente, & Maciel‐de‐Freitas, 2016). Nevertheless, to minimize the impact of bloodfed status on our microbiota characterization, we excluded visibly bloodfed females from this study, and thus feel that the contribution of trap type to error variation in vector microbiota is minimal. Finally, in this study, we wanted to characterize the breadth of microbes associated with a species, and therefore, pooling of individuals was appropriate. Although pooling resulted in the loss of information at the individual mosquito level, others have observed individuals to be highly variable in field‐caught specimens (Osei‐Poku et al., 2012).

In characterizing microbiota diversity across vectors, we acknowledge that sample preparatory steps including the use of phenolic extraction and differing PCR protocols may have an impact on our data. A previous study indicated that the PCR conditions that we used could introduce biases in the next‐generation sequencing, including a higher risk of errors and chimera production (Ahn, Kim, Song, & Weon, 2012). To limit biases introduced by PCR, we performed a strict sequence quality control. In the future, the use of mock community control, although not common practice at the time of this study, is recommended to quantify sequence contamination (Lusk, 2014; Salter et al., 2014). However, the latter study also show that contamination is more pronounced with samples containing low microbial biomass. As mosquitoes are known to harbor high titers of bacteria and eukaryotic microbes (Minard et al., 2013), we believe that contamination contributed minimally to our data.

In this study, the most important factor affecting mosquito microbiota was vector species, and secondarily habitat. In spite of variation in microbial diversity and composition between samples, we observed vector‐specific clustering based on weighted UniFrac distances for both *Cx*. *quinquefasciatus* and *Aedes* pools, driven largely by abundant vector‐specific taxa *Wolbachia* (*Ae. albopictus* and *Cx. quinquefasciatus*) and *Ascogregarina* (*Aedes*). *Ascogregarina taiwanensis* (Apicomplexa: Lecudinidae) and *A. culicis* are protozoan parasites of *Ae. albopictus* and *Ae. aegypti*, respectively (Lantova & Volf, 2014), although cross‐infections can occur between larvae (Lantova & Volf, 2014). The observed differences between diversity indices of microbiota between vector species could be influenced by the ability of vector‐specific symbionts such as *Wolbachia* and *Ascogregarina* to colonize their hosts in high number, as suggested in another study (Minard et al., 2014). However, we expect that richness indices, rather than diversity indices that measure relative abundance, are more robust to the “saturation” effect of *Wolbachia* or *Ascogregarina* read numbers.

Thus, vector microbiota includes common obligate endosymbionts that tend to be vector‐specific, as well as rare and/or facultative microbes whose distribution is heterogeneous across vectors but may depend in part on habitat. Indeed, for a given vector species, microbiota varied across different habitats. Mosquitoes are thought to acquire their microbiota from the environment in which the species develops and lives (although some bacteria, such as *Wolbachia*, are also vertically transmitted). For example, mosquitoes acquire *Ascogregarina* sp. from their larval habitat (Lantova & Volf, 2014). Many other bacteria are shared within and between species through the larval aquatic habitat (Coon et al., 2016). As a result, the diversity and composition of a vector's microbiota are determined by, at least partially, the diversity and composition of microbes in their habitat, which can in turn be highly variable (Coon et al., 2016). In our study, composition and diversity in terms of rarefaction asymptote estimates of OTU richness, indices of diversity, and number of identified taxa, particularly for bacteria, showed an increasing trend in diversity from urban to rural sites. This trend may be due to greater complexity in terms of heterogeneity of rural habitats relative to urban sites, supporting more microbe species, by various potential mechanisms for species distributions within and across landscapes (Tscharntke et al., 2012). In contrast, a study of field‐caught mosquitoes from Ghana found that midgut microbiota was more diverse in urban mosquitoes compared to rural ones (Akorli et al., 2016). However, Akorli et al. (2016) sampled sites that were much farther apart and in markedly different ecological zones (forest‐savannah transitional zone vs. coastal savannah zone) compared to our study. Thus, differences they observed in mosquito microbiota could be as much a function of ecological zone as urbanization.

The results of this study suggest that in addition to biodiversity loss occurring with land‐use change and habitat degradation in terms of mosquito diversity (Thongsripong et al., 2013), similar patterns may exist at the microbial level, in terms of the microbiota of mosquitoes. Loss of microbial diversity has been observed in other habitats modified by humans and in some cases associated with increased disease risk. A shift in soil microbial communities has been observed when forest is transformed to pasture (Nüsslein & Tiedje, 1999); reduced soil microbial diversity has also been reported in arable land (Torsvik, Ovreas, & Thingstad, 2002). Agricultural transformation of land also favors pathogenic *Burkholderia pseudomallei* in soil communities (Kaestli et al., 2009); this can include higher abundance at sites in agricultural versus unfarmed land (Limmathurotsakul et al., 2010). Notably, ecological differences in the distributions of certain mosquitoes, such as *Ae. aegypti*, an invasive species which increases relative to *Ae. albopictus* as habitats become increasingly modified by humans (Thongsripong et al., 2013), will also affect the distribution of specific microbial agents such as *Wolbachia*, species of which occur natively in *Ae. albopictus* and are known to interfere with infectious agents such as dengue, chikungunya, and Zika viruses. Thus, shifts in the communities of nonpathogenic vector‐associated microbes in response to ecological changes and distribution of vectors could affect the distribution of pathogens across human‐modified landscapes. The relationship between increased threats to human health via infectious microbes and land‐use change and/or biodiversity loss has been reported in several systems, linked to urbanization, agricultural intensification, and deforestation (McFarlane, Sleigh, & McMichael, 2013; Olson, Gangnon, Silveira, & Patz, 2010; Vitter et al., 2006; Wilcox & Colwell, 2005). Our results highlight the importance of subsequent investigation to determine how symbiotic microbial diversity of mosquito vectors responds to habitat change and the implications for human health.

Because most female mosquitoes feed on vertebrates as a requisite for egg maturation, we wanted to explore the impact of vertebrate diversity on microbiota diversity and composition. Others have shown that mosquito midgut bacterial communities are dynamic and vary significantly with respect to bloodfed state (Wang et al., 2011). Host DNA is detectable by sequencing even when engorgement is no longer visible (Davey, Casey, Burgess, & Cable, 2007; Lee et al., 2002), and quite interestingly, we identified *18S rRNA* sequences belonging to phylum Chordata, class Mammalia, in a few of our mosquito samples. Although their presence was not frequent enough across pools to conduct further analyses, with the everimproving depth of sequencing provided by technological advances, we envision future studies being able to simultaneously characterize both microbial community composition and vertebrate host utilization from field‐collected mosquitoes.

## 5 CONCLUSIONS

This field study characterizes both bacterial and eukaryotic microbiota associated with naturally occurring mosquito vector species using culture‐independent methods. The diversity of microbiota associated with mosquitoes collected in our study differed both between vector species and across environments within a given species. Importantly, for *Ae. aegypti*, microbiota diversity decreased along a gradient of increasing habitat modification from rural to urban, warranting further study to confirm this finding and to investigate the role of microbial symbiont diversity in vector‐borne disease transmission. We found the composition of mosquito microbiota to be largely vector‐specific and often dominated by a few taxa, such as *Ascogregarina* and *Wolbachia*. Our multidomain metabarcoding approach also revealed potential bacterial and microbial eukaryote interactions within the vector host. This study is a first step in improving our understanding of mosquito microbiota, elements of which (e.g., *Wolbachia*) are known to influence disease transmission. Further research is required to understand how the dynamics of mosquito microbiota across vectors and landscapes can affect mosquito‐borne disease transmission, and ultimately the impact of land use and associated habitat change on infectious disease emergence.

## DATA ACCESSIBILITY

Raw unprocessed sequencing reads are available through the NCBI Short Read Database as part of BioProject PRJNA406988, and the representative sequences of the bacterial and eukaryotic datasets are found in Tables S5 and S6, respectively.

## CONFLICT OF INTEREST

None declared.

## AUTHORS' CONTRIBUTIONS

PT participated in study design, mosquito identification, molecular laboratory experiment, and sequence analysis and carried out fieldwork. JAC participated in sequence analysis. ABG participated in study design and mosquito identification and carried out fieldwork. PK participated in study design and coordinated and accommodated field experiments and parts of molecular laboratory processes. BAW, DDK, and SNB conceived of the study and participated in study design and coordination. PT, JAC, and SNB wrote the manuscript. DDK made substantial edits. All authors read, gave input, and approved the final manuscript.
