# Supplementary material for: Mosquito vector‐associated microbiota: Metabarcoding bacteria and eukaryotic symbionts across habitat types in Thailand endemic for dengue and other arthropod‐borne diseases
Source: Ecol Evol. 2017 Dec 27;8(2):1352–68. doi: 10.1002/ece3.3676 (PMC5773340; doi:10.1002/ece3.3676)
Supplement: Supplementary file 1 [file ECE3-8-1352-s001.docx]

**Supplementary Figures**

**Supplementary Figure 1**. Command lines for sequence analysis using QIIME

#QIIME analysis for 16s rRNA sequences

split_libraries.py -m map.file.16s.txt -f G8477TI02.fasta -q G8477TI02.qual -b 11 –o full.QC.noTruncate/ -e 0 -w 50 –a 0 –H 9

denoise_wrapper.py -i G8477TI02.sff.txt -f 16s_split_seqs.fna -o 16sDenoised/ -m map.file.16s.txt –titanium

inflate_denoiser_output.py -c centroids.fasta -s singletons.fasta –d denoiser_mapping.txt -o denoised_16s.fasta -f 16s_split_seqs.fna

truncate_reverse_primer.py -f denoised_16s.fasta -m map.file.16S.txt -o denoised_truncated_16s/ -z truncate_remove -M 1

pick_de_novo_otus.py -i denoised_16ssta_rev_primer_truncated.fna -o otus_16s/

filter_otus_from_otu_table.py -i otu_table.biom -o filtered_table.biom --negate_ids_to_exclude

-e denoised_16ssta_rev_primer_truncated_rep_set_aligned.fasta

convert_biom.py -i filtered_table.biom -o filtered_16s_table.txt -b --header_key taxonomy --process_obs_metadata taxonomy

identify_chimeric_seqs.py -m ChimeraSlayer -i denoised_16ssta_rev_primer_ truncated_rep_set_aligned.fasta -o chimeric_16s_2.txt –a //macqiime/greengenes/core_set_ aligned.fasta.imputed

filter_fasta.py -f denoised_16ssta_rev_primer_truncated_rep_set.fasta -o non_chimeric_rep_set_aligned.fasta -s chimeric_16s.txt –n

filter_otus_from_otu_table.py -i filtered_table.biom –o chimera_filtered_table.biom –e chimeric_16s_2.txt

convert_biom.py -b --header_key taxonomy --process_obs_metadata taxonomy -i chimera_filtered_16s_table.biom -o chimera_filtered_16s_table.txt

# QIIME analysis for 18s rRNA sequences

split_libraries.py -m map.file.18s.txt -f G8477TI02.fasta -q G8477TI02.qual -b 11 -o full.QC.noTruncate/ -e 0 -w 50 –a 0 –H 9

denoise_wrapper.py -i G8477TI02.sff.txt -f 18s_split_seqs.fna -o 18sDenoised/ -m map.file.18s.txt --titanium

inflate_denoiser_output.py -c centroids.fasta -s singletons.fasta -d denoiser_mapping.txt -o denoised_18s.fasta -f 18s_split_seqs.fna

truncate_reverse_primer.py -f denoised_18s.fasta -m map.file.18S.txt -o denoised_truncated_18s/ -z truncate_remove -M 1

pick_otus.py -i denoised_18ssta_rev_primer_truncated.fna -o 18s_otu

pick_rep_set.py -i denoised_18ssta_rev_primer_truncated_otus.txt

-f denoised_18ssta_rev_primer_truncated.fna -o rep_set_18s.fna

assign_taxonomy.py -i rep_set_18s.fna -t Silva_RDP_taxa_mapping.txt

-r rep_set/silva_104_rep_set.fasta -o assigned_tax_18s/

make_otu_table.py -i denoised_18ssta_rev_primer_truncated_otus.txt

-t rep_set_18s_tax_assignments.txt -o otu_table_18s.biom

convert_biom.py -b --header_key taxonomy --process_obs_metadata taxonomy

-i otu_table_18s.biom -o otu_table.18s.txt

align_seqs.py -i rep_set_18s.fna -t core_aligned_set/core_Silva_aligned.fasta -o pynast_aligned_18s/

filter_alignment.py -i pynast_aligned_18s/rep_set_18s_aligned.fasta -o pynast_aligned_18s_2/ -e 0.10 -g 0.80

make_phylogeny.py -i pynast_aligned_18s_2/rep_set_18s_aligned_pfiltered.fasta -o /rep_set_18s.tre

filter_otus_from_otu_table.py -i otu_table_18s.biom -o filtered_table_18s.biom

--negate_ids_to_exclude -e pynast_aligned_18s_2/rep_set_18s_aligned_

pfiltered.fasta

convert_biom.py -b --header_key taxonomy --process_obs_metadata taxonomy

-i filtered_table_18s.biom -o filtered_table_18s.txt

identify_chimeric_seqs.py -m ChimeraSlayer -i pynast_aligned_18s/rep_set_18s_aligned.fasta -a core_aligned_set/core_Silva_aligned.fasta -o chimeric_seqs_18s.txt

filter_fasta.py -f rep_set_18s.fna -o 18s_non_chimeric_rep_set_aligned.fasta -s chimeric_seqs_18s.txt -n

filter_otus_from_otu_table.py -i filtered_table_18s.biom -o chimera_filtered_18s_table.biom -e chimeric_seqs_18s_2.txt

convert_biom.py -b --header_key taxonomy --process_obs_metadata taxonomy -i chimera_filtered_18s_table.biom -o chimera_filtered_18s_table.txt

**Supplementary Figure 2.** Rarefaction curves indicating richness of *Aedes albopictus*-associated bacterial and eukaryotic OTUs derived from 16s rRNA (a) and 18s rRNA (b). The expected numbers of bacterial and eukaryotic OTUs associated with *Aedes albopictus* collected in rural habitats for three different pool sizes, 25, 9 and 3 individual mosquitoes plotted as a function of sampling effort (in this case, number of sequences).

a

b

**Supplementary Figure 3.** Non-metric Multidimensional Scaling (NMDS) plots showing the differences in bacterial (top figure) and eukaryotic microbial (bottom figure) composition in different species of mosquito pools derived from multiple habitats. The analysis was performed using R’s package Vegan V2.4-4 (metaMDS function with the default choice monoMDS to perform global NMDS).


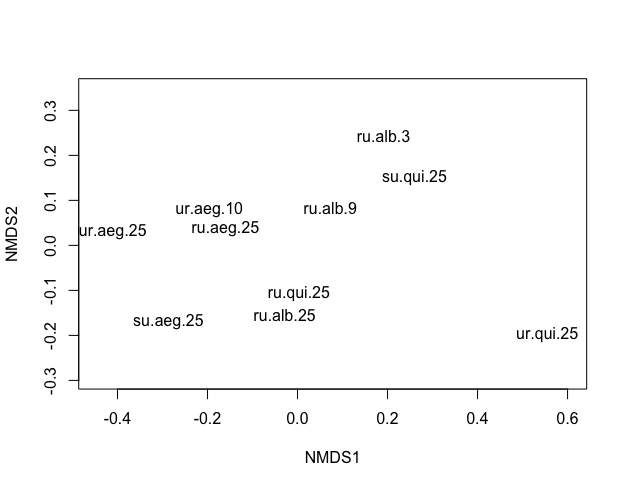


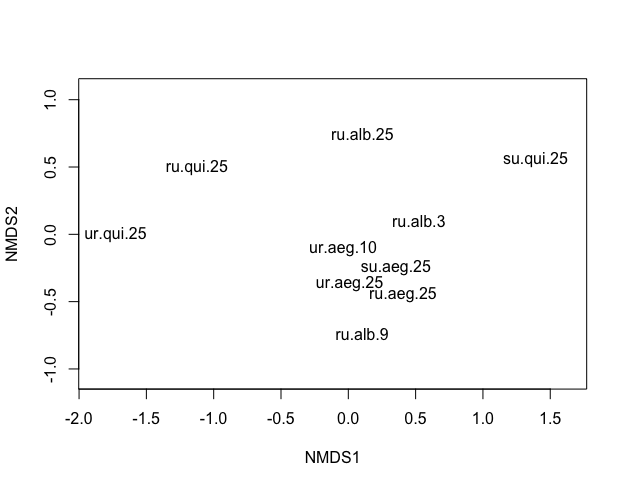


**Suppliementary Figure 4.** Clustering (weighted Unifrac) after excluding *Wolbachia* (16s rRNA sequence, upper figure) and *Ascogregarina* (18s rRNA sequence, lower figure) sequences.


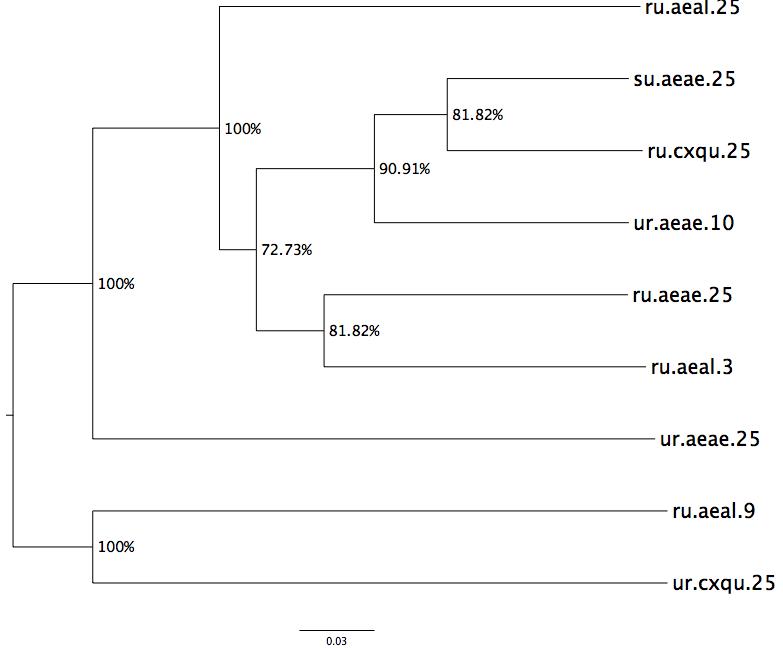


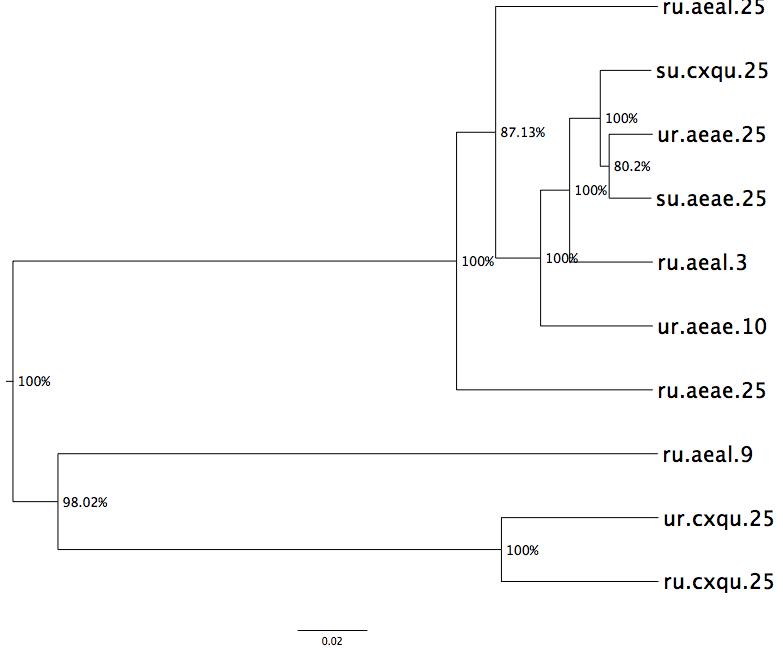


**Supplementary Figure 5.** Phylogenetic relationship between *Wolbachia* OTUs identified in this study compared to representative taxa. Maximum likelihood (ML) phylogenetic tree is based on 16S rRNA of study reads (subsampled) as well as publically available reference sequences. ML bootstrap support values are shown at nodes. *Aedes aegypti* derived reads are shown in red.
